# In situ synthesis of ZnO/g-C_3_N_4_ based composites for photodegradation of methylene blue under visible light

**DOI:** 10.1038/s41598-024-84645-0

**Published:** 2025-01-02

**Authors:** S. Pourali, R. Amrollahi, S. Alamolhoda, S. M. Masoudpanah

**Affiliations:** 1 School of Metallurgy and Materials Engineering, Iran University of Science and Technology, Narmak, Tehran, Iran; 2 Department of Physics, Iran University of Science and Technology, Narmak, Tehran, Iran

**Keywords:** Methylene blue, Photocatalytic activity, Visible light, ZnO/g-C_3_N_4_ based composites, Photocatalysis, Synthesis and processing

## Abstract

In this study, in situ-synthesized ZnO/g-C_3_N_4_ based composites were used as photocatalysts for organic pollution removal. These nanocomposites were prepared through simple calcination of a mixture of melamine and ZnO nanoparticles and underwent comprehensive evaluation of their structural, morphological, optical, and photocatalytic properties, using various analytical techniques. As the g-C_3_N_4_ content increased, the band gap decreased from 3.02 to 2.94 eV. Additionally, the reduction in photoluminescence intensity confirmed the heterojunction interface between the g-C_3_N_4_ and ZnO components. The photodegradation rate of methylene blue (MB) dye exhibited an increase, rising from 0.016 (min^−1^) for ZnO and 0.011 (min^−1^) for g-C_3_N_4_ to 0.022 (min^−1^) for the ZnO/g-C_3_N_4_ (10 wt%) composite. Furthermore, combining ZnO (50 wt%) with g-C_3_N_4_ led to a significant enhancement in the MB dye removal efficiency, reaching 97% compared to the ZnO/g-C_3_N_4_ (10 wt%) composite. In contrast, the removal efficiencies were 90% for pristine ZnO and 73% for g-C_3_N_4_ phases.

**Supplementary Information:**

The online version contains supplementary material available at 10.1038/s41598-024-84645-0.

## Introduction

The rapid pace of economic development in recent decades has resulted in significant environmental challenges, particularly due to the increasing release of harmful pollutants that contaminate water and air. This underscores the urgent need for research into simple and cost-effective pollutant removal technologies^1–7^. Methylene blue (MB), a widely used industrial dye, serves as an ideal model pollutant for evaluating photocatalytic performance due to its ease of detection in real-world applications. MB is particularly concerning due to its toxicity, carcinogenicity, and resistance to biodegradation, posing serious threats to human health and the environment. Its presence in natural water sources exacerbates these risks, highlighting the critical need for eco-friendly and efficient technologies to eliminate MB from wastewater. Photocatalysis has emerged as a promising approach for MB removal^8^.

Among various photocatalysts, ZnO, a metal oxide semiconductor, has attracted significant attention, especially in the past decade. It has been used in many applications, such as photocatalysts, light-emitting diodes, optical sensors, and electron detectors^9,10^. ZnO is favored for its high electron mobility, good photochemical performance, non-toxic nature, low cost, and high redox potential^10,11^. However, ZnO has some limitations, such as being active only under UV light (which constitutes just 4% of the solar spectrum), rapid recombination of electron-hole pairs, and susceptibility to corrosion under light, which limits its stability and photocatalytic activity. Various strategies have been proposed to address these limitations^10,12–14^.

In recent years, g-C_3_N_4_, a non-metallic semiconductor, has gained attention for its ability to degrade organic pollutants, produce hydrogen, and reduce CO_2_ under visible light irradiation^15,16^. g-C_3_N_4_ is cost-effective, readily available, and possesses suitable electronic properties and high stability, particularly under visible light. It also exhibits high thermal and chemical stability in aqueous solutions and in acidic and alkaline environments due to the strong covalent bonds between carbon and nitrogen atoms. However, g-C_3_N_4_ faces challenges such as high electron-hole recombination rates, weak electrical conductivity, limited oxidation ability of holes in the valence band, and low quantum yield^17,18^. Researchers have explored various strategies to enhance the photocatalytic properties of g-C_3_N_4_, including creating porous structures, introducing metallic or non-metallic dopants, coupling with graphene, organic linking, and forming nanocomposites with other nanomaterials^7,19^.

Coupling ZnO with g-C_3_N_4_ has been identified as a promising solution to improve the separation efficiency of light-generated carriers and enhance oxidation-reduction capabilities. This method provides an appropriate band structure for the two materials, leading to the formation of spatial charge accumulation at the interface, which facilitates the separation of photo-generated electron-hole pairs. Additionally, this approach allows for the utilization of a broader light spectrum for photocatalytic activation^20,21^. Traditional methods such as mixing/calcination^22^, milling^23^, ultrasonic dispersion^24^, and hydrothermal synthesis^25^ often result in non-uniform distribution of ZnO in ZnO/g-C_3_N_4_ nanocomposites, reducing the reliability and repeatability of the synthesis for practical applications^26^. In contrast, our in-situ synthesis via simple calcination ensures a more uniform distribution of ZnO nanoparticles in the g-C_3_N_4_ matrix. This method not only simplifies the preparation process but also enhances the structural integrity and photocatalytic performance of the nanocomposites. Our approach provides significant improvements in synthesis efficiency, scalability, and cost-effectiveness.

In this study, spherical ZnO nanoparticles were initially synthesized using the solution combustion method. Subsequently, their composite with g-C_3_N_4_ was prepared via a straightforward calcination process, marking the first instance of such a preparation method. This approach offers simplicity and cost-effectiveness while ensuring excellent recyclability. Furthermore, the impact of varying g-C_3_N_4_ doping ratios on the photocatalytic effects was systematically investigated, and a detailed analysis of the photocatalytic reaction mechanism was conducted.

## Experimental procedures

### Materials

Analytical grades of the starting materials were purchased from Merck GmbH, including zinc nitrate hexahydrate (Zn(NO_3_)_2_·6H_2_O), citric acid (C_6_H_8_O_7_), melamine (C_3_H_6_N_6_), and sodium hydroxide (NaOH). These substances were used to prepare the essential solutions for the experiments.

### Sample preparation

#### Synthesis of zinc oxide nanoparticles

Zinc oxide nanoparticles were synthesized using zinc nitrate as the zinc precursor and citric acid as the fuel, following Eq. (1) with the optimal ratio of 1:1. The mixture was heated on a hotplate stirrer at about 80 °C until a gel was formed. The gel was transferred to a preheated aluminum container, which induced self-combustion. This process resulted in a white zinc oxide powder, which was then ground and homogenized in a mortar.

$$ \text{Zn}(\text{NO}_{3} )_2+  \frac{5}{9} \text{C}_6 \text{H}_8 \text{O}_7  +  \frac{5}{2}  \text{O}_2 \rightarrow \text{ZnO} +  \frac{30}{9}  \text{CO}_2 + \frac{20}{9}  \text{H}_2 \text{O}+ \text{N}_2$$

#### Synthesis of graphitic carbon nitride

Graphitic carbon nitride was prepared by thermal condensation of melamine. Briefly, 10 g of melamine was placed in a crucible covered with an aluminum foil sheet in a box furnace at a temperature of 550 ℃ for 2 h with a heating rate of 20 ℃/min. Finally, the products were cooled in the furnace^27^.

#### Synthesis of ZnO/g-C_3_N_4_ nanocomposite

**Fig. 1:**
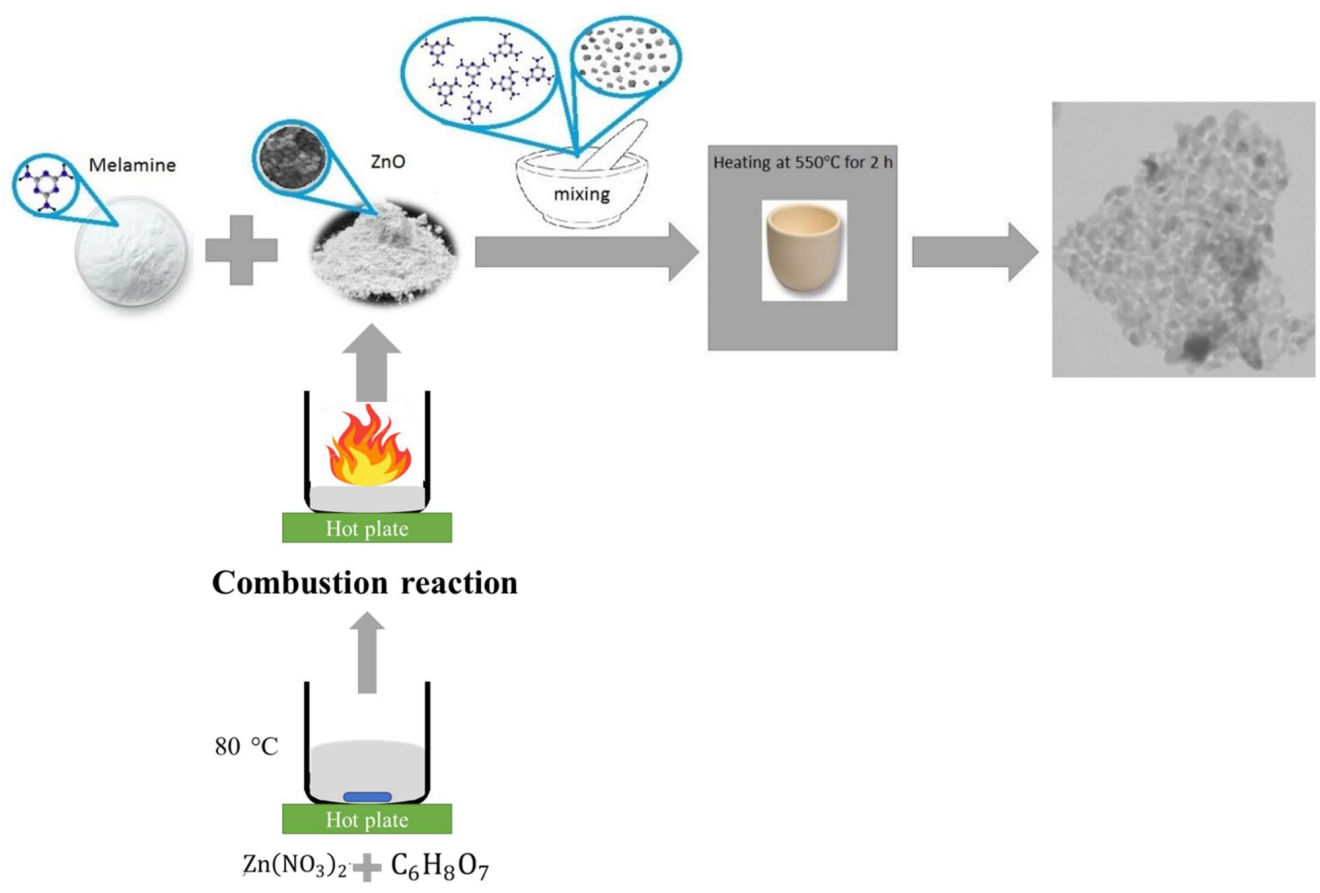
Schematic illustration of the in-situ synthesis process of ZnO/g-C_3_N_4_ nanocomposite.

ZnO/g-C_3_N_4_ nanocomposite was prepared by mixing and grinding zinc oxide (ZnO) with melamine in different weight ratios (10:90, 25:75, 33:67, 50:50). The mixture was homogenized in a mortar for 15 min and then calcined in a sealed alumina crucible at 550 ℃ for 2 h with a heating rate of 20 ℃/min. Photocatalytic tests were performed on the composite after it was cooled down in the furnace. The synthesis process of ZnO/g-C_3_N_4_ nanocomposite is illustrated in Fig. 1.

### Photocatalytic experiment

The photocatalytic performance of the nanocomposites was evaluated using 0.1 g of the synthesized ZnO/g-C_3_N_4_ nanocomposites with 100 mL of MB dye at concentrations of 5, 10, and 15 ppm, and pH levels of 5, 7, 10, and 13. The pH of the solution was adjusted using NaOH. Photocatalytic experiments were conducted with two 100 W xenon lamps, which provide a broad spectrum of light similar to natural sunlight. The spectral range of the xenon lamps covers both UV and visible regions, closely matching the wavelength distribution of natural sunlight, ensuring that the observed photocatalytic performance under laboratory conditions is representative of real-world applications.

The suspension was stirred in the dark for 60 min to reach adsorption-desorption equilibrium, then exposed to visible light irradiation. Every 60 min, 5–7 mL of the suspension was sampled and centrifuged at 4000 rpm for 20 min to separate the solid and liquid phases. The concentration of the degraded solution was measured using a UV-vis spectrophotometer. MB dye was chosen as a model pollutant because it is widely used in textile industries and has a strong absorption peak in the visible region.

### Characterization

The X-ray diffraction (XRD) patterns of the synthesized samples were recorded by a Philips X’Pert device, using monochromatic CuKα radiation with a wavelength of 0.15418 nm at a goniometer angle of 20 to 80 degrees. The optical absorption of the samples was measured by a UV-vis spectrophotometer in a VARIO 2600 device. A Shimadzu UV-vis-52550 device is used for the diffuse reflectance spectroscopy (DRS) analysis in the range of 200–800 nm. The Fourier transform infrared spectroscopy (FTIR) spectra of the samples were recorded by a Thermo Avatar FTIR device in the range of 400–4000 cm^−1^. The morphology of the samples was analyzed by a field emission, scanning electron microscope (FESEM) (MIRA3TESCAN) and a transmission electron microscope (TEM) (Philips cm300) with an operating voltage of 20 kV. An ASAP micromeritics 2020 device was used for N_2_ adsorption/desorption to analyze the surface area, pore volume, and pore size distribution of the samples. The photoluminescence (PL) analysis of the samples was evaluated by a Hitachi F-7000 Fluorescence device with an excitation wavelength of 325 nm. A mixture of ZnO/melamine (50:50) was analyzed by STA (BäHR 503, Germany) instrument in air atmosphere using heating rate of 20 °C/min.

## Results and discussion

### Material characterization

**Fig. 2:**
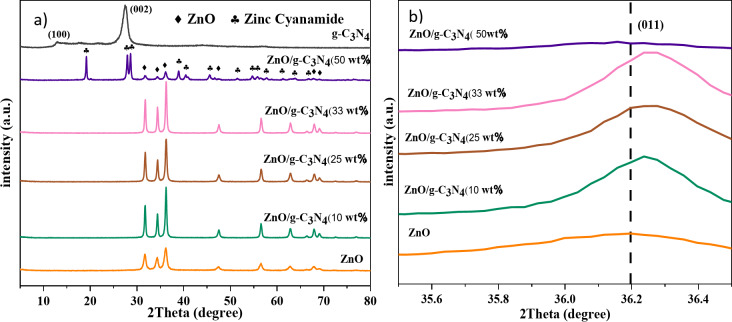
(**a**) X-ray diffraction pattern of ZnO, g-C_3_N_4_ and g-C_3_N_4_/ZnO composites, (**b**) Enlarged X-ray diffraction pattern of ZnO and g-C_3_N_4_/ZnO composites.

The XRD patterns of the samples are presented in Fig. 2. Two distinct peaks at 27.38° and 12.74° were observed in the XRD pattern of the g-C_3_N_4_, which can be attributed to the (002) and (100) planes, respectively. The strong peak at 27.38° corresponds to the interlayer spacing of 0.334 nm, related to the stacking of the aromatic sp^2^-hybridized systems. The weak peak at 12.74° corresponds to the interlayer spacing of 0.685 nm, derived from the in-plane structural arrangement of the tri-s-triazine units (Fig. 2a)^18^.

In the XRD pattern of the ZnO/g-C_3_N_4_ composites (ZnO/g-C_3_N_4_ based composite), almost all the characteristic diffraction peaks of hexagonal wurtzite ZnO were observed, confirming the presence of ZnO phase. In particular, the diffraction peak of ZnO in the nanocomposite shifted slightly to higher angles with an increasing weight% of g-C_3_N_4_ (10, 25, 33), indicating that the addition of g-C_3_N_4_ affects the electron cloud density of ZnO (Fig. 2b). The ZnO/g-C_3_N_4_ (50 wt%) showed a two-phase composition of ZnO and zinc cyanamide, such that the intensity of the diffraction peaks of ZnO decreased after the combination with g-C_3_N_4_. The relatively weak crystallization of ZnO phase indicates that the simultaneous crystallization of g-C_3_N_4_ phase interferes with the stacking and alignment of ZnO phase patterns, resulting in weak scattering^21,28^.

The absence of noticeable diffraction peaks of g-C_3_N_4_ species in the XRD patterns of ZnO/g-C_3_N_4_ composite is due to the strong bonding of g-C_3_N_4_ during alloying with ZnO, which is likely achieved through a condensation reaction between the amino triazine group and the hydroxyl groups on the surface of ZnO to form Zn-N bond^29^. As a result, the van der Waals force between the graphitic C_3_N_4_ sheets is broken, causing the loss of intensity of its peaks during the reaction^30^.

**Table 1:** Crystallite size and band gap values for ZnO, g-C_3_N_4_ and ZnO/g-C_3_N_4_ composites.

Sample	D_XRD_ (nm)	Eg (eV)
ZnO	19.3	3.05
g-C_3_N_4_	9.4	2.60
ZnO/g-C_3_N_4_ (10 wt.%)	26.5	3.02
ZnO/g-C_3_N_4_ (25wt.%)	26.5	2.98
ZnO/g-C_3_N_4_ (33wt.%)	30.3	2.96
ZnO/g-C_3_N_4_ (50wt.%)	41.6	2.94

As shown in Table 1, the average crystallite size in the composite samples increased significantly compared to ZnO, indicating the effect of g-C_3_N_4_ substrate on the crystallization of ZnO^10^.

**Fig. 3:**
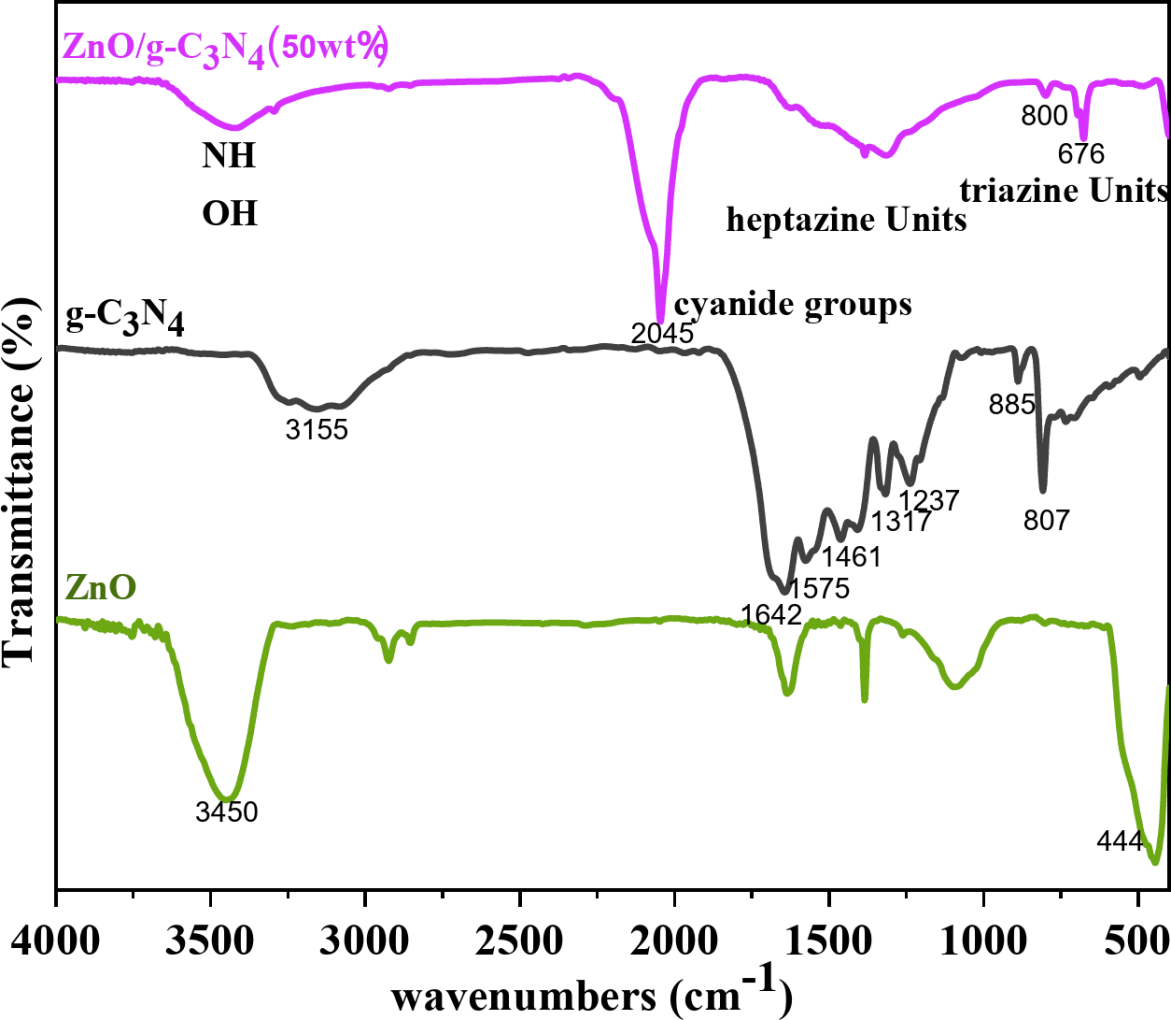
FTIR spectra of ZnO, g-C_3_N_4_ and ZnO/g-C_3_N_4_ (50 wt.%) samples.

The functional groups and the chemical bonds of ZnO, g-C_3_N_4_, and ZnO/g-C_3_N_4_ (50 wt%) were identified by FTIR spectrum, and the results are highlighted in Fig. 3. In the FTIR spectrum of g-C_3_N_4_, several strong bands were found in the region of 1237–1642 cm^−1^, corresponding to the stretching modes of typical CN heterocycles. The peaks at 1642, 1461, and 1557 cm^−1^ were assigned to the stretching modes of repeating units derived from heptazine. The peaks at 1237 and 1317 cm^−1^ were attributed to the stretching modes of triangular units connected by C-N(-C)-C or C-NH-C (partial condensation)^19^. The characteristic peak at 807 cm^−1^ was assigned to the out-of-plane bending modes of triazine units, which are related to condensed CN heterocycles. The absorption band at 885 cm^−1^ matched with the deformation mode of N-H in amino groups. The broad absorption band around 3100–3400 cm^−1^ was related to water molecules adsorbed and non-condensed N-H groups on the surface of g-C_3_N_4_^21,31^. The FTIR spectrum of ZnO showed a peak at 444 cm^−1^, which was related to the stretching vibrations of the Zn-O bond. Due to the absorption of water molecules in the oxide surface, a peak at 3450 cm^−1^ was observed, corresponding to the hydroxyl absorption peak^32^.

The FTIR spectrum of the ZnO/g-C_3_N_4_ (50 wt%) was similar to the characteristic bands of g-C_3_N_4_, indicating the graphitic carbon nitride structure, which was well preserved after homogeneous hybridization with ZnO. The composite showed a red shift with weakened intensity in the characteristic bands of g-C_3_N_4_, indicating that a conjugated system containing g-C_3_N_4_ and ZnO was produced instead of simple physical adsorption^28^.

Compared to pure g-C_3_N_4_, the characteristic peaks of the stretching modes of C-N heterocycles in the range of 1237–1642 cm^−1^ were merged into a broad absorption band in the sample ZnO/g-C_3_N_4_ (50 wt%), indicating the effect of ZnO crystallization. The emergence of a new peak at 2045 cm^−1^ indicated the presence of attached cyanide groups, which were used as intermediates during the polymerization of melamine. The appearance of cyanide groups in the ZnO/g-C_3_N_4_ (50 wt%) showed that the simultaneous crystallization of ZnO could interfere with the thermal polymerization of melamine to produce the g-C_3_N_4_ phase. Such interference of ZnO crystallization could result in relatively weak crystallinity and increase the defects in the g-C_3_N_4_ phase^21^.

**Fig. 4:**
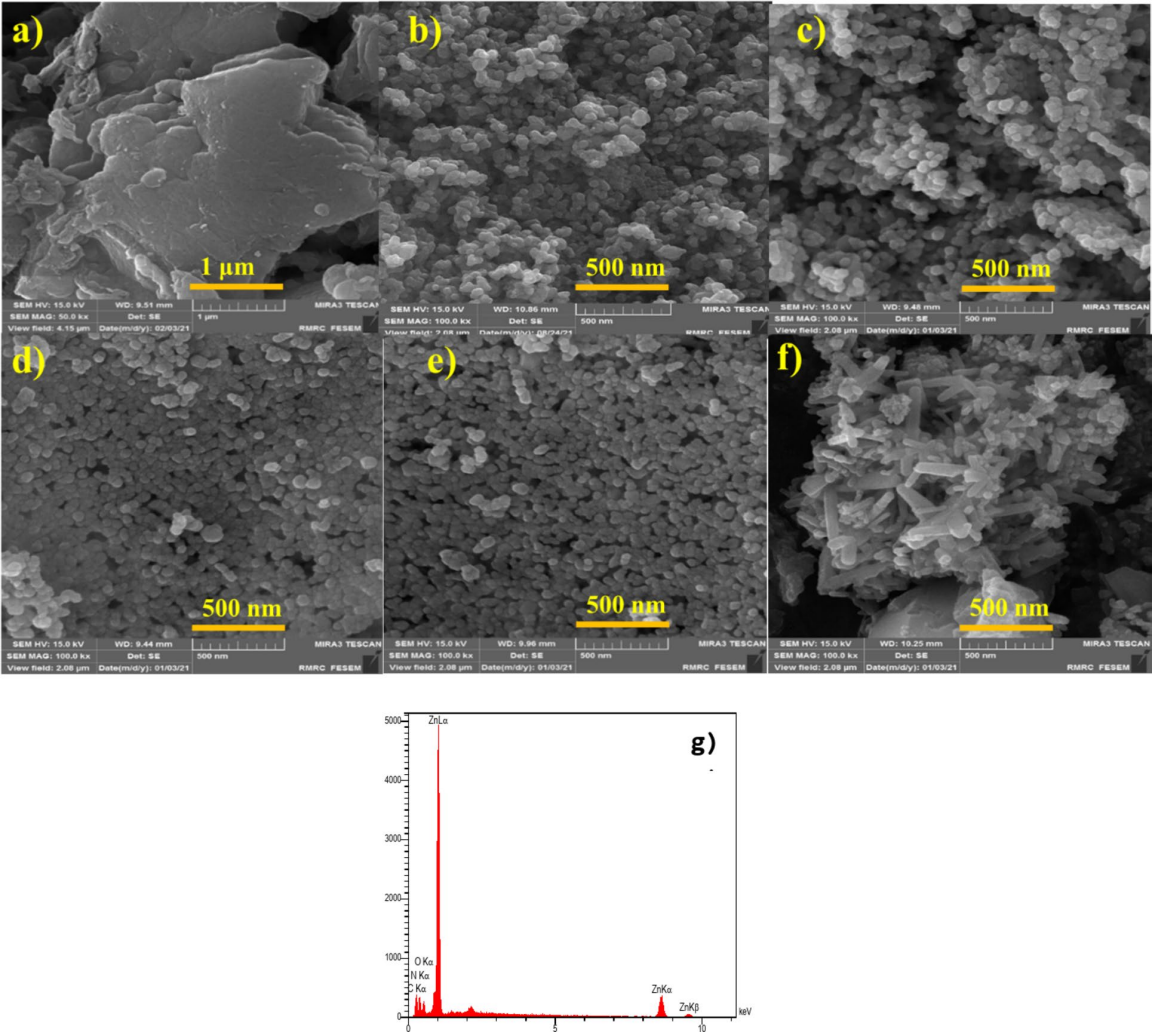
FESEM image of (**a**) g-C_3_N_4_, (**b**) ZnO, (**c**) ZnO/g-C_3_N_4_(10 wt%), (**d**) ZnO/g-C_3_N_4_(25 wt%), (**e**) ZnO/g-C_3_N_4_ (33 wt%), (**f**) ZnO/g-C_3_N_4_ (50 wt%) nanocomposites and (**g**) EDX image of ZnO/g-C_3_N_4_ (50 wt%) nanocomposite.

The morphology of the samples was investigated using a scanning electron microscope. Figure 4 shows the images of pure graphitic carbon nitride and ZnO/g-C_3_N_4_ nanocomposite with different weight ratios (10, 25, 33, 50 wt%). Pure g-C_3_N_4_ consists of stacked graphitic sheets, which are made of tri-s-triazine building units (Fig. 4a). The composites ZnO/g-C_3_N_4_ (10, 25, 33 wt%) (Fig. 4c–e) are mainly agglomerated spheres with an average size of about 50 nm, which are composed of many small spherical nanoparticles, probably due to the densifying role of melamine in the chemical deposition process. Meanwhile, the addition of g-C_3_N_4_ to ZnO (pure ZnO is shown in Fig. 4b) did not result in any significant change in the overall morphology. In addition, it was difficult to distinguish between ZnO and g-C_3_N_4_ particles, which might be attributed to the coating of g-C_3_N_4_ on the surface of ZnO particles^33–35^.

The mesoporous layered structure characteristic of g-C_3_N_4_ can be well preserved in the final nanocomposite even after hybridization with ZnO. As such, the ZnO/g-C_3_N_4_ (50 wt%) composite has a porous sponge-like structure composed of multiple nanoparticles (Fig. 4e), distinct from the morphology of pure ZnO and g-C_3_N_4_. This may be due to the effect of gases released from precursor decomposition and condensation during high-temperature calcination in air. In addition, the nanocomposite structure is much denser than pure ZnO. Such elimination of high porosity and conversion to denser structures is mainly due to the crystallization process of the g-C_3_N_4_ phase embedded within the primary pores of ZnO^21,28^. This combination results in interfacial solid contact between ZnO and g-C_3_N_4_ and increases the lifetime of photo-generated electron-hole pairs, thereby reducing internal charge recombination. This is very beneficial for improving photocatalytic activity. EDX analysis was performed to determine the chemical composition and purity of the ZnO/g-C_3_N_4_ (50 wt%). Figure 4g pinpoints that C, N, Zn and O elements were uniformly distributed in the nanocomposite with weight percentages of 22.46%, 31.95%, 35.86%, and 9.73%, respectively. The uniform distribution of elements indicates that ZnO and g-C_3_N_4_ phases were nicely mixed and closely contacted in the nanocomposite instead of aggregating separately^21,36^.

**Fig. 5:**
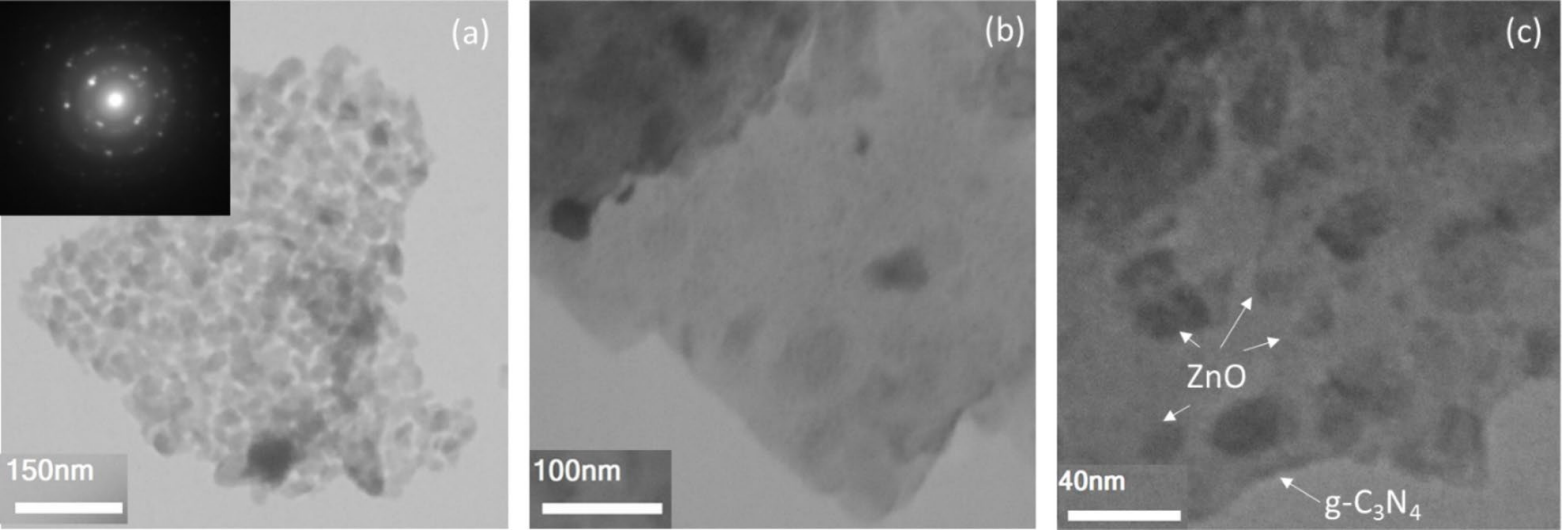
TEM images and the related SAED pattern of the ZnO/g-C_3_N_4_ (50 wt%) nanocomposite.

TEM micrographs of ZnO/g-C_3_N_4_ (50 wt%) and the related selected area diffraction (SAED) pattern is indicated in Fig. 5a–c. As can be seen, hybrids ZnO/g-C_3_N_4_ (50 wt%) consist of spherical ZnO nanoparticles, randomly and densely distributed on the surface of g-C_3_N_4_ sheets.

In conclusion, XRD analysis reveals that the formation of ZnO/g-C_3_N_4_ heterojunctions significantly affects the crystal structure, which is evident from the shift in diffraction peaks. FTIR spectra confirm the strong chemical bonding between ZnO and g-C_3_N_4_, which is crucial for efficient charge transfer. SEM and TEM images show the uniform distribution of ZnO nanoparticles on g-C_3_N_4_ sheets, which enhances light absorption and reduces recombination rates. UV-vis spectroscopy demonstrates the extended absorption edge in the visible region, correlating with the enhanced photocatalytic activity.

**Fig. 6:**
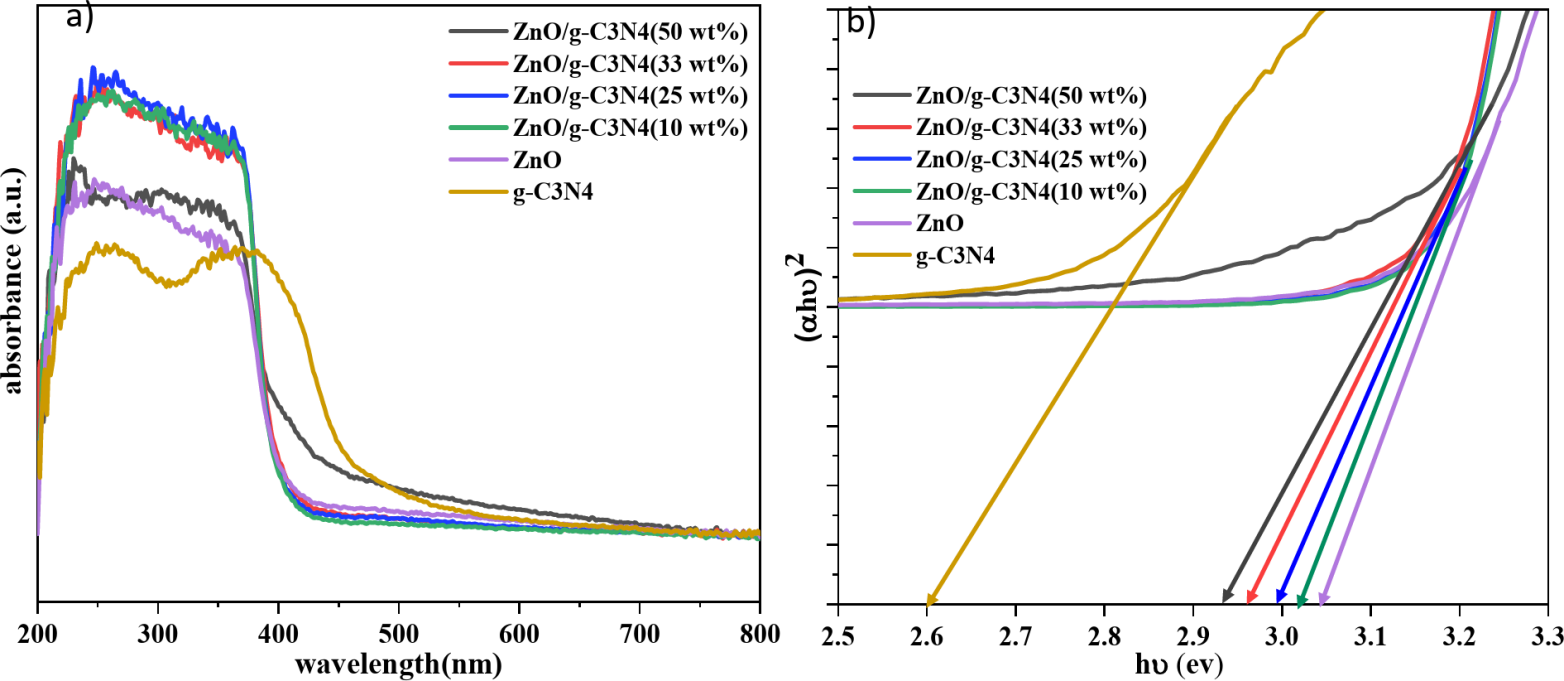
(**a**) Absorption spectrum, (**b**) Plot of (αhυ)^2^ versus photon energy for ZnO, g-C_3_N_4_ and g-C_3_N_4_/ZnO composites.

The photoabsorption behavior of the prepared composite photocatalysts was investigated, using UV-vis spectroscopy (Fig. 6a,b). Pure wurtzite ZnO oxide has a strong absorption edge at 427 nm, corresponding to a band gap of 3.05 eV, which well demonstrates that ZnO is a semiconductor material with excellent photoelectric properties in the ultraviolet regions but gives relatively poor response to visible light. In contrast to the absorption behavior of ZnO, the absorption edge of g-C_3_N_4_ occurs at 483 nm, creating a band gap of 2.60 eV, which indicates its photocatalytic activity induced by visible light^28,32^.

Meanwhile, as shown in Fig. 6a, at high loading of g-C_3_N_4_, the absorption edges of the composites shift significantly to a longer wavelength region compared to pure ZnO, showing stronger and broader absorption than itself. These results may be attributed to the strong chemical bonding interaction via the interface between g-C_3_N_4_ and ZnO phases in the composite samples. This observation also indicates that adding g-C_3_N_4_ changes the reflectance intensity of photocatalysts ZnO/g-C_3_N_4_, which means higher absorption and broader utilization of visible light. Given the calculated band gaps shown in Table 1, with increasing amounts of g-C_3_N_4_, the optical band gaps of nanocomposites decrease from 3.02 to 2.94 eV. These results indicate that the absorption edge of composites move toward lower energy region by coupling ZnO and g-C_3_N_4_. This enhances the light response by the material. The resulted rapid charge transfer and more production of photo-generated electron-hole pairs under visible light irradiation, subsequently leads to higher photocatalytic activity. The diffuse reflectance spectroscopy (DRS) results show that synthesized samples can absorb in visible and UV regions, which can be promising for photocatalysts in solar light^28,33,37^.

**Fig. 7:**
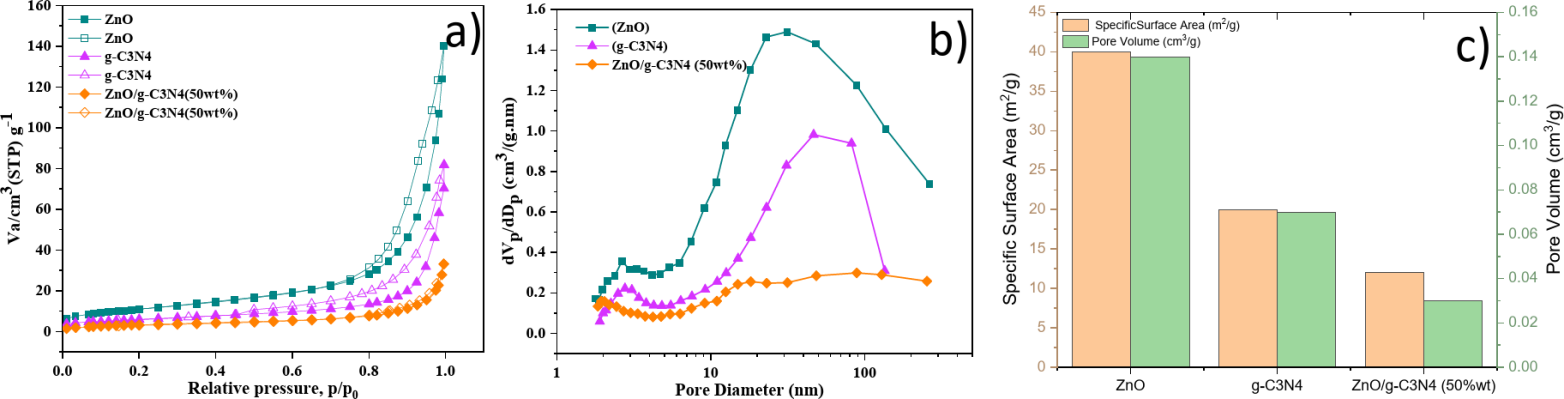
(**a**) Adsorption (solid symbols) and desorption (empty symbols) isotherms related to ZnO, g-C_3_N_4_, ZnO/g-C_3_N_4_ (50 wt%) samples, (**b**) BJH pore size distribution, (**c**) Specific surface area and pore volume of the samples.

The adsorption-desorption isotherms obtained using nitrogen gas were used to determine the textural properties of pure g-C_3_N_4_, ZnO, and ZnO/g-C_3_N_4_ (50 wt%). The results are shown in Fig. 7a–c, which according to IUPAC classification, indicate isotherm type IV with H_3_ hysteresis loop, indicating the presence of mesoporous structure (2–50 nm). In general, pores are formed due to the aggregation of nanoparticles. Mesopores play an essential role in adsorption for catalysts in solid-liquid systems. The calculated parameters of physical properties, including specific surface area, pore volume, and average pore diameter are highlighted in Table 2. Pure ZnO (40 m^2^.g^−1^) has a higher specific surface area than obtained ZnO/g-C_3_N_4_ (50 wt%) (20 m^2^.g^−1^), indicating that surface area is not the main factor for increasing photocatalytic performance. g-C_3_N_4_ changes overall in the entire relative pressure range upwardly.

**Table 2:** Specific surface area, pore volume and pore size values for ZnO, g-C_3_N_4_ and ZnO/g-C_3_N_4_ (50wt.%) samples.

Sample	Specific surface area (m^2^/g)	Pore volume (cm^3^/g)	Mean pore diameter (nm)
ZnO	40	0.14	14.4
g-C_3_N_4_	20	0.07	13.6
ZnO/g-C_3_N_4_ (50wt.%)	12	0.03	9.8

The significant reduction of specific surface area and pore volume indicates that ZnO crystallization can fill the primary pores in the g-C_3_N_4_ phase, leading to an increased interfacial contact degree between g-C_3_N_4_ and ZnO phases^21,36^. Comparison of pore size distribution also confirms that ZnO/g-C_3_N_4_ (50 wt%) has fewer mesopores and a smaller pore volume than pure ZnO. The results presented in Table 2 calculated by Barrett–Joyner–Halenda (BJH) method also indicate that pure ZnO has a larger pore volume and pore diameter than the composite.

The photocatalytic activity of ZnO/g-C_3_N_4_ nanocomposites with various weight percentages (10, 25, 33, and 50 wt%) was examined under visible light, using an organic dye (MB). Prior to activating the light source, an adsorption-desorption equilibrium was established by immersing the MB aqueous solution with the prepared photocatalysts in darkness for 60 min, while continuous stirring was maintained.

**Fig. 8:**
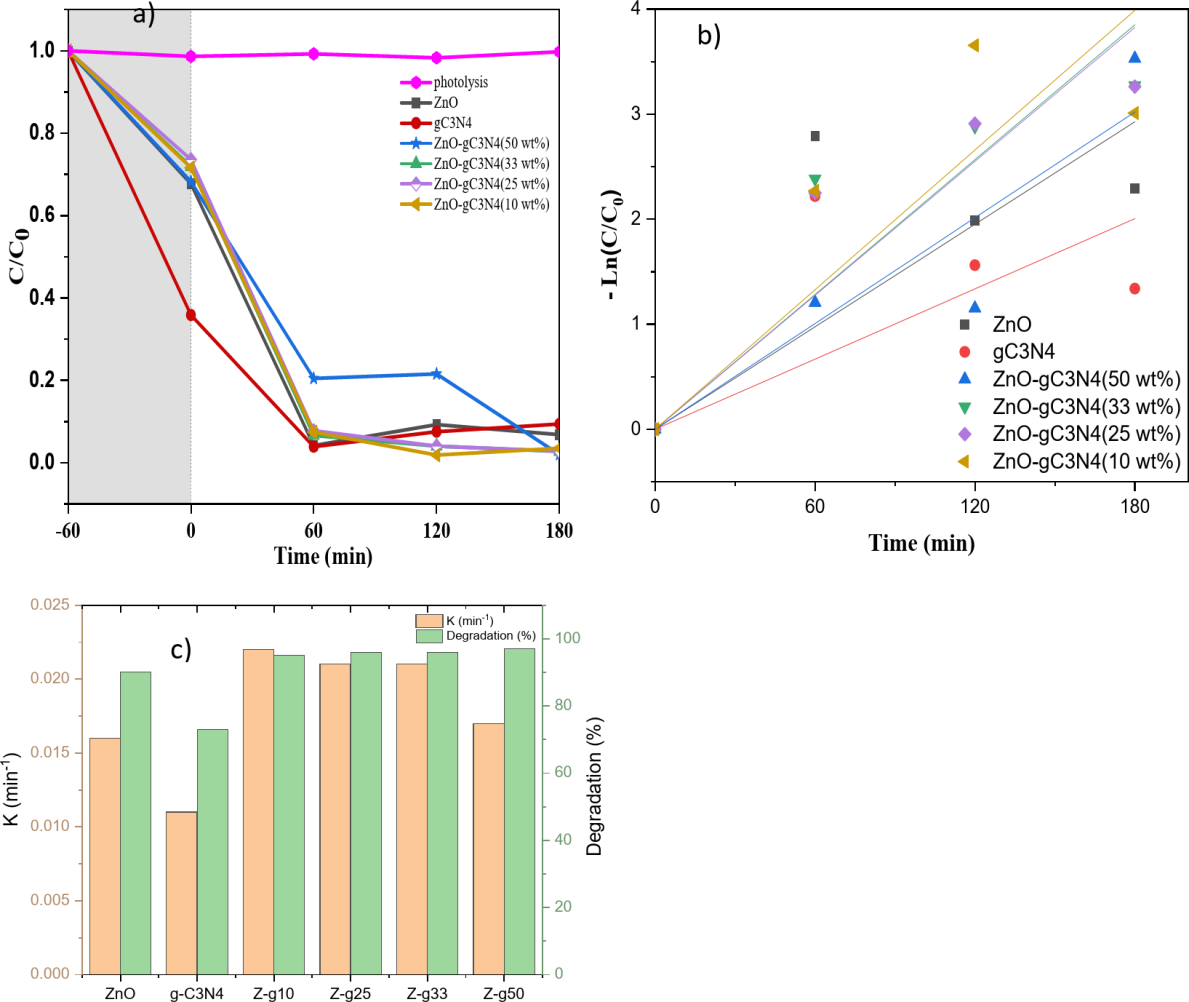
(**a**) C/C_0_ and (**b**) ln (C/C_0_) vs. irradiation time for photodegradation of MB. (**c**) Rate constant diagram of ZnO, g-C_3_N_4_ and ZnO/g-C_3_N_4_ composites under visible light irradiation.

Figure 8 highlights the changes in MB concentration under visible light irradiation in the presence of ZnO, g-C_3_N_4_, and ZnO/g-C_3_N_4_ nanocomposites at pH 10. In a controlled experiment, methylene blue (MB) underwent photolysis to assess dye degradation in the absence of a catalyst. The results revealed that MB degradation during the irradiation period was minimal, suggesting that MB molecules remain stable under visible light exposure without a catalyst. However, when MB was irradiated in the presence of g-C_3_N_4_, a noticeable reduction in concentration occurred. This highlights the catalytic effect of g-C_3_N_4_ on MB photodegradation. Interestingly, significant changes in concentration of organic dye molecules are observed in the presence of ZnO/g-C_3_N_4_ nanocomposites. Additionally, the total organic carbon (TOC) removal percentage of MB after irradiation in the presence of ZnO/g-C_3_N_4_ was reported to be 93%, confirming light-induced MB degradation^38^. Studies have determined that the intermediates formed by photodegradation include azure A, azure B, azure C, and thionine, which eventually degrade completely into CO_2_ and H_2_O^38,39^.

The degradation efficiency and the photocatalytic rate constant evaluated are shown in Fig. 8a–c. ZnO shows relatively weak activity (i.e., degradation efficiency = 90%) under visible light irradiation. Structural and electronic defects, such as interstitial oxygen and oxygen vacancies during synthesis process extend absorption to higher wavelength and reduce band gap energy. Therefore, higher defect level increases trapping ability of photo-generated charge carriers (electron-hole pairs) and improves photocatalytic performance^40^. In addition, improper orientation of lattice planes along spherical particles with random distribution of pores increases strain and absorption under visible region. The porous nature of spherical ZnO nanoparticles exposes more sites for photocatalytic reaction, which is an additional advantage during photolytic analysis^41^. g-C_3_N_4_ adsorbs 64% of MB molecules in darkness, showing a significant reduction in concentration.

However, 60 min after irradiation, the MB concentration slightly increased, which can be attributed to the re-adsorption of MB molecules, so g-C_3_N_4_ degraded about 73% of the MB molecules in 180 min of irradiation. The degradation efficiency for ZnO/g-C_3_N_4_ (10, 23, 33, 50 wt%) increased to 95, 96, 96 and 97%, respectively. The photocatalytic activity of ZnO/g-C_3_N_4_ nanocomposites increased proportionally with the g-C_3_N_4_ content in the composites. Remarkably, the ZnO/g-C_3_N_4_ composite containing 50 wt% g-C_3_N_4_ exhibited the highest photocatalytic activity, achieving an impressive light degradation efficiency of 97%. Furthermore, the rate constant for the ZnO/g-C_3_N_4_ composite (50 wt%) (0.021 min^−1^) was approximately 1.5 times higher than that observed for pure zinc oxide (0.013 min^−1^). However, further reduction of g-C_3_N_4_ amount to 10 wt% resulted in a decrease in the degradation rate. It is also hypothesized that an excess of ZnO bulks can serve as recombination sites for photogenerated hole-electron pairs, potentially impeding the charge transfer induced by light^28^. The observed degradation result confirms that g-C_3_N_4_ significantly improves the photocatalytic activity. In general, more reactive species, larger surface area, and rich active sites can enhance the photocatalytic activity. The synthesized powders with higher specific surface area adsorb more dyes on their surface and have more reaction sites for degradation^42^.

### Effect of solution pH

**Fig. 9:**
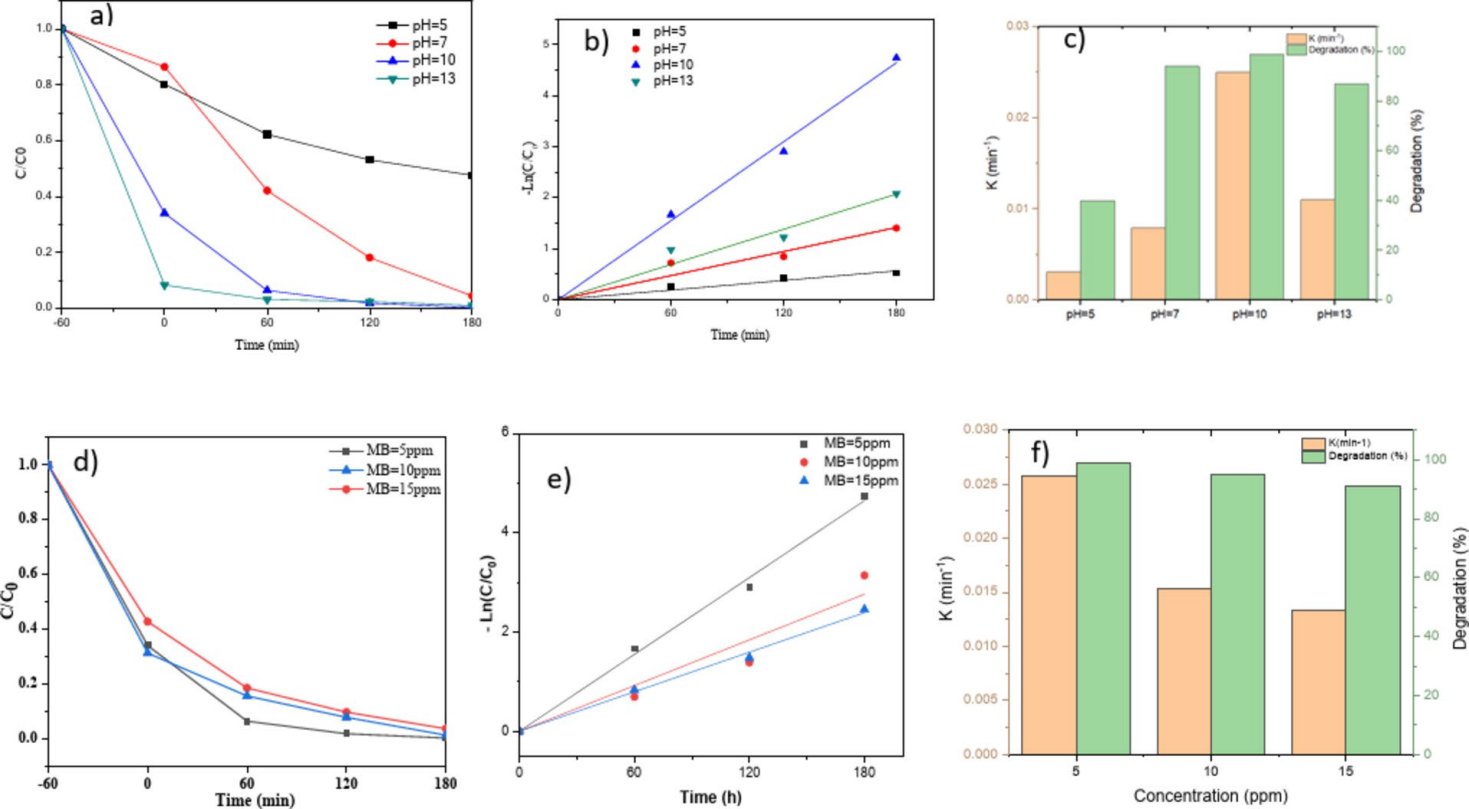
(**a**) Effect of pH change on photocatalytic degradation of MB dye (5 ppm) for ZnO/g-C_3_N_4_ (50 wt%) nanocomposites, and (**b**,**c**) Reaction rate constant and degradation percentage of MB dye at different pH values for ZnO/g-C_3_N_4_ (50 wt.%) nanocomposite, (**d**) effect of dye concentration on photocatalytic degradation of MB for ZnO/g-C_3_N_4_ (50 wt%), (**e**,**f**) Reaction rate constant and degradation percentage of methylene blue dye at different concentrations.

Figure 9a–c indicates the degradation curves of MB dye as a function of irradiation time for ZnO/g-C_3_N_4_ (50 wt%) at pHs of 5, 7, 10 and 13 and the related rate constants. By changing the pH in the solution, the charge on the catalyst surface changes. At a specific pH, the catalyst surface charge is neutral, called the point of zero charge (PZC). For zinc oxide, PZC is equal to 9. The expected surface reaction at different pH values is shown in Eqs. (2) and (3)^33,43^.

2 $$ \text{pH} \le \text{pH}_\text{PZC};\quad \text{ZnOH} + \text{H}^{+}  \rightarrow \text{ZnOH}_{2}^{+} $$

3 $$ \text{pH} \ge \text{pH}_\text{PZC};\quad \text{ZnOH} + \text{OH}^{-}  \rightarrow \text{ZnO}^{-} + \text{H}_{2}\text{O} $$

The MB dye is cationic and in this kind of dye, with increasing pH, the degradation rate increases. In an acidic solution, the photocatalyst surface develops a positive charge, thus preventing MB adsorption to its surface. The maximum degradation occurs when visible light irradiates to ZnO/g-C_3_N_4_ (50 wt%) at pH 10 and in 180 min of irradiation.

The degradation rate of the dye was increased with the addition of ZnO catalyst, which could be attributed to the enhanced adsorption of the dye molecules on the catalyst surface. The higher photocatalytic activity resulted from the electrostatic attraction between the positively charged dye molecules and the negatively charged ZnO surface. On the other hand, by increasing pH the amount of hydroxyl ion increases, which plays a significant role in degradation^33,36^. When pH increased from 10 to 13, the degradation percentage decreased again, and color removal decreased from 99 to 87%. In an alkaline environment, ZnO surface is negatively charged, and MB usually acts as its un-ionized neutral form. Therefore, Na cations may compete with MB molecules for available active sites in this condition, reducing photocatalytic activity^33^. Figure 9c shows the reaction rate constants and dye degradation percentages at different pHs for ZnO/g-C_3_N_4_ (50 wt%).

### Effect of dye concentration

Dye concentration significantly influences photocatalytic color removal. The experiments were conducted using MB dye at concentrations of 5, 10, and 15 ppm. Figure 9d–f pinpoints the degradation of different concentrations of methylene blue dye as a function of time for ZnO/g-C_3_N_4_ (50 wt%) at pH 10. The research conducted by Davis et al. reveals an inverse correlation between dye concentration and the path length of incoming photons within the solution. Consequently, lower dye concentrations result in fewer available photons for absorption by the catalyst. Given this study, the optimal dye concentration for this system is 5 ppm. Figure 9f illustrates the reaction rate constant and dye degradation percentage at pH 10^36^.

**Fig. 10:**
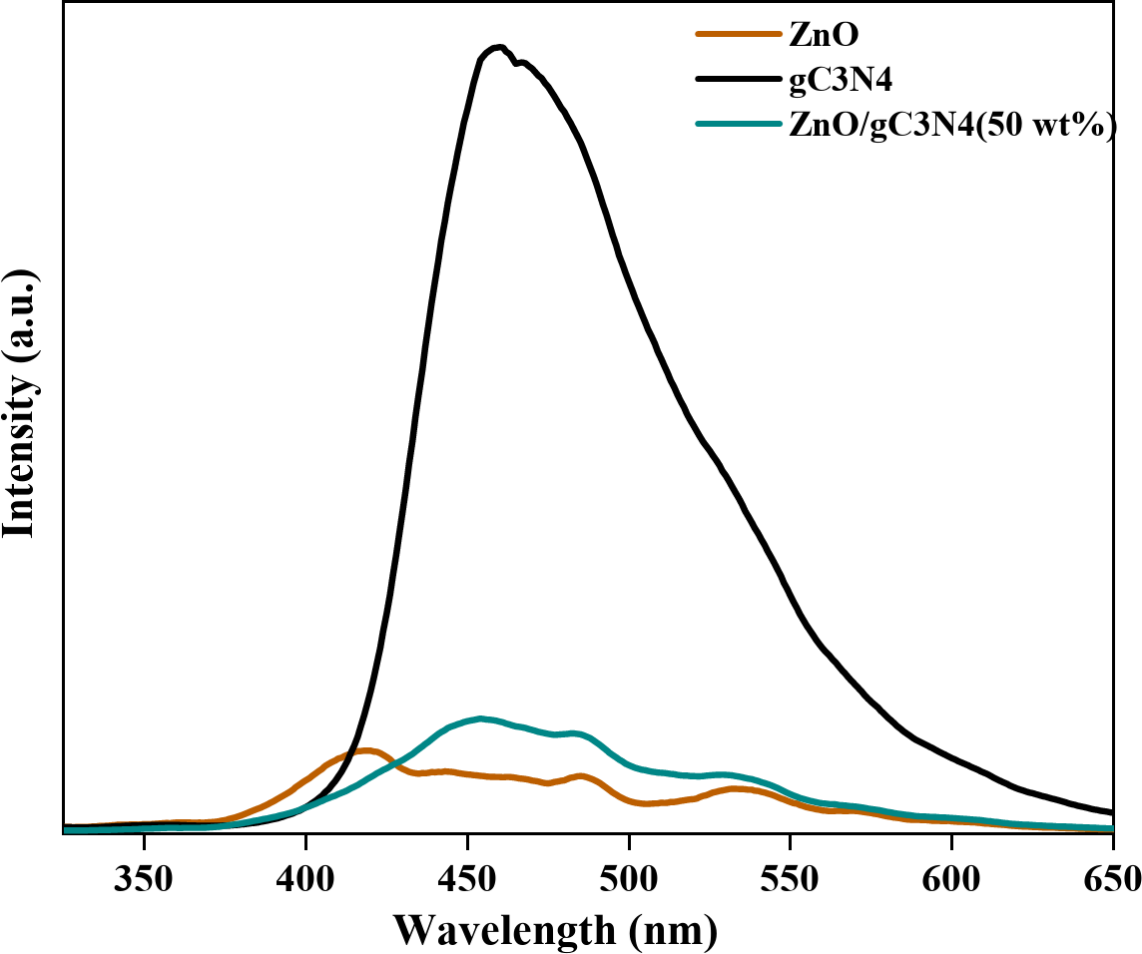
PL spectra of ZnO, g-C_3_N_4_ and ZnO/g-C_3_N_4_ (50 wt%) samples.

To investigate the behaviors of separation and transfer of free charge carriers and further photocatalytic performance of catalysts, PL spectroscopy measurements, which result from the recombination of photo-generated electrons and holes are used. The PL emission spectra for ZnO, g-C_3_N_4_, and ZnO/g-C_3_N_4_ (50 wt%) are highlighted in Fig. 10 at room temperature.

It is observed that the central emission peak for pure g-C_3_N_4_ appears at around 460 nm, which is equivalent to the energy of the band gap of pure g-C_3_N_4_ (2.7 eV), resulting from the intrinsic n-π* electronic transitions^10,38^.

Pure ZnO involves three emission peaks at 420 nm, 485 nm, and 530 nm, possibly due to the surface oxygen vacancies and defects in ZnO. In contrast, the ZnO/g-C_3_N_4_ nanocomposite simultaneously shows the intrinsic fluorescence emission peaks of ZnO, and g-C_3_N_4_ and the emission intensity decreases, indicating that the photo-induced electron-hole pairs are effectively suppressed^18^.

When ZnO absorbs light at a wavelength of 325 nm, electrons from the valence band move to the conduction band, leaving behind holes. These transferred electrons in the conduction band eventually recombine with the holes in the valence band, releasing energy and resulting in PL emission. The higher observed PL intensity indicates a faster recombination rate of photo-induced electron-hole pairs in the g-C_3_N_4_ sample.

In the case of the ZnO/g-C_3_N_4_ nanocomposite, photo-induced electrons transfer from the g-C_3_N_4_ to the ZnO conduction band. This transfer occurs because the conduction band edge potential of g-C_3_N_4_ (−1.12 eV) is more negative than that of ZnO (−0.2 eV). Simultaneously, holes move from the ZnO valence band to the g-C_3_N_4_ valence band. This separation and efficient transfer of charge carriers within the ZnO/g-C_3_N_4_ nanocomposite effectively suppresses recombination processes, resulting in lower PL intensity and enhanced photocatalytic activity^18^.

**Fig. 11:**
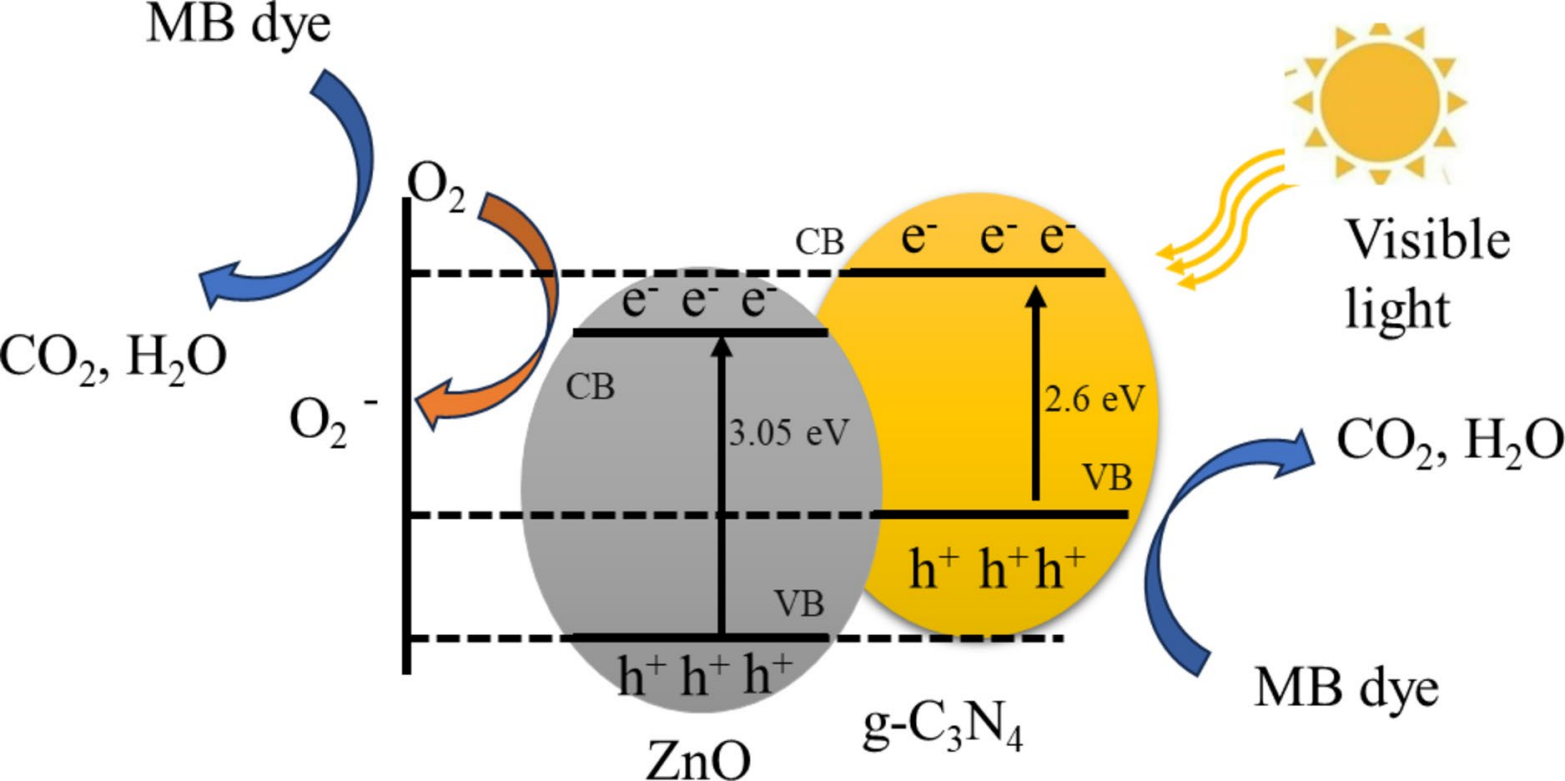
Overview of the energy band structure and electron-hole pair separation in the ZnO/g-C_3_N_4_ nanocomposite.

The formation of a heterojunction between ZnO and g-C_3_N_4_ plays a crucial role in facilitating charge separation^10,18^. The band alignment at the interface creates a built-in electric field that drives the photogenerated electrons from g-C_3_N_4_ to ZnO and holes in the opposite direction, effectively reducing recombination rates. This is supported by the observed reduction in photoluminescence intensity and the enhanced photocatalytic activity. The band alignment diagram (Fig. 11) illustrates the charge transfer mechanism across the heterojunction interface.

We have conducted thorough stability evaluations, including TGDT and scavenger tests under optimal conditions. The detailed findings from these experiments can be found in the Supplementary Material. Furthermore, we have performed XRD analysis on the catalyst both before and after reuse to assess its structural integrity and any potential alterations resulting from repeated use (Supplementary Figs. 1, 2, 3 and 4).

## Conclusion

The study investigated the impact of graphitic carbon nitride (g-C_3_N_4_) on the photocatalytic performance of in situ prepared ZnO/g-C_3_N_4_ nanocomposites under visible light irradiation. X-ray diffraction (XRD) patterns revealed characteristic peaks of hexagonal wurtzite (ZnO) in the ZnO/g-C_3_N_4_ (10–33 wt%) composites, while the ZnO/g-C_3_N_4_ (50 wt%) composite exhibited a two-phase composition of ZnO and zinc cyanamide. Transmission electron microscopy (TEM) micrographs depicted spherical ZnO nanoparticles densely distributed on the surface of g-C_3_N_4_ sheets.

The presence of g-C_3_N_4_ significantly enhanced the photocatalytic performance of ZnO/g-C_3_N_4_, leading to increased visible light absorption and a narrowed band gap energy. Specifically, the optical band gaps of the nanocomposites decreased from 3.02 to 2.94 eV with increasing g-C_3_N_4_ content. Photoluminescence (PL) spectroscopy measurements indicated lower emission intensity for the ZnO/g-C_3_N_4_ (50 wt%) nanocomposite compared to pure ZnO, suggesting reduced electron-hole recombination induced by light in the composite.

Furthermore, the photodegradation rate of methylene blue (MB) dye improved from 0.016 min^−1^ (for ZnO) and 0.011 min^−1^ (for g-C_3_N_4_) to 0.022 min^−1^ for the ZnO/g-C_3_N_4_ (10 wt%) composite at pH 10, using a 5 ppm dye concentration. The removal efficiency of MB dye increased from 95 to 97% for ZnO/g-C_3_N_4_ (50 wt%) and ZnO/g-C_3_N_4_ (10 wt%) composites, while pristine ZnO and g-C_3_N_4_ phases achieved removal efficiencies of approximately 90% and 73%, respectively.

## Electronic supplementary material

Below is the link to the electronic supplementary material.

Supplementary Material 1

## Data Availability

All data generated or analysed during this study are included in this published article.
