# Supplementary material for: In situ synthesis of ZnO/g-C3N4 based composites for photodegradation of methylene blue under visible light
Source: Sci Rep. 2025 Jan 2;15:462. doi: 10.1038/s41598-024-84645-0 (PMC11696719; doi:10.1038/s41598-024-84645-0)
Supplement: Supplementary file 1 — Supplementary Material 1 [file 41598_2024_84645_MOESM1_ESM.docx]

**Supplementary Material:**

**In situ synthesis of ZnO/g-C_3_N_4_ based composites for photodegradation of methylene blue under visible light**

S. Pourali^a^, R. Amrollahi^*b^, S. Alamolhoda^a^, S. M. Masoudpanah^a^

*Corresponding Author’s Email Address: [amrollahir@iust.ac.ir](mailto:amrollahir@iust.ac.ir)

a. School of Metallurgy & Materials Engineering, Iran University of Science and Technology, Narmak, Tehran, Iran

b. Department of Physics, Iran University of Science and Technology, Narmak, Tehran, Iran

**Stability of the ZnO/gC₃N₄ photocatalyst:**

To evaluate the practical applicability of the synthesized ZnO/g-C_3_N_4_ composite, its stability and reusability were tested across four cycles of methylene blue (MB) degradation under visible light irradiation. After each photocatalytic cycle, the spent photocatalyst was thoroughly rinsed with distilled water and ethanol, dried at 80°C for 2 hours, and reused under the same conditions for subsequent cycles. The photocatalytic degradation efficiency remained consistently high throughout all reuse cycles (97 ± 2%), indicating minimal loss of activity. Even after four consecutive cycles, only a slight decrease in MB degradation was observed, confirming the excellent stability of the ZnO/g-C_3_N_4_ photocatalyst (Supplementary Figure 1).

This remarkable stability can be attributed to the composite’s robust and homogeneous structure, which promotes the efficient migration of photogenerated electrons and holes from the conduction band (CB) and valence band (VB) to the surface of the photocatalyst, thereby reducing the likelihood of degradation. The XRD patterns of the recycled catalyst obtained before and after the photocatalytic reaction are also shown in the Supplementary Figure 2. This clearly indicates that the composite catalyst is stable during the reaction.

Supplementary Figure 1: Normalized concentration of MB over time during photocatalytic degradation by ZnO/ g-C_3_N_4_

Supplementary Figure 2: XRD pattern of the ZnO/g-C_3_N_4_ photocatalyst before and after the photocatalytic degradation of MB dye solution.

**DTA analysis:**

DTA analysis of the ZnO/melamine mixture shows an endothermic peak at approximately 350 °C, followed by an exothermic peak around 440 °C. ZnO has a reported melting point of 1975 °C [1], while melamine undergoes sublimation and condensation between 300 and 390 °C [2]. As the DTA test was conducted in an air atmosphere, the observed exothermic peak may be attributed to the decomposition of the formed g-C_3_N_4_. TGA results reveal a weight loss of approximately 75% within the temperature range of 300 to 450 °C. Our previous TGA studies have indicated that g-C_3_N_4_ begins to decompose around 600 °C, whereas in metal oxide/g-C_3_N_4_ composites, this weight loss occurs at lower temperatures (Supplementary Figure 3). This reduction in decomposition temperature could be due to the high number of crystal defects in the g-C_3_N_4_ species generated during composite preparation [3].

Supplementary Figure 3: DTA analysis of the ZnO/melamine mixture.

**Effect of radical scavengers on the photocatalytic degradation of methylene blue:**

To elucidate the role of reactive species in the photocatalytic degradation of methylene blue (MB), radical trapping experiments were conducted at various pH levels. Specifically, 1 mM solutions of isopropyl alcohol (IPA), benzoquinone (BQ), and ammonium oxalate (AO) were added to the MB solution to quench hydroxyl radicals (OH^•^), superoxide radicals (O₂^•⁻^), and holes (h⁺), respectively. As depicted in Supplementary Figure 4, the addition of AO, IPA, and BQ scavengers significantly inhibited the photodegradation of MB under visible light irradiation in the presence of the ZnO/ g-C_3_N_4_ (50 wt%) nanocomposite. These findings suggest that while hydroxyl radicals, superoxide radicals, and holes all contribute to the photocatalytic process, superoxide radicals (O₂^•⁻^) play the most dominant role in the MB degradation by the nanocomposite.

Supplementary Figure 4: Effect of radical scavengers on the photocatalytic degradation of methylene blue (MB) by ZnO/g-C_3_N_4_ (50 wt%) under visible light irradiation at pH 10 (right) and pH 7 (left).

**References:**

[1] R. Triboulet, "Growth of ZnO bulk crystals: A review." Progress in Crystal Growth and Characterization of Materials 60.1 (2014): 1-14.

[2] G. Dong, Y. Zhang, Q. Pan, J. Qiu, A fantastic graphitic carbon nitride (g-C_3_N_4_) material: Electronic structure, photocatalytic and photoelectronic properties, Journal of Photochemistry and Photobiology C: Photochemistry Reviews 20 (2014) 33–50.

[3] M. Afkari, S. M. Masoudpanah , M. Hasheminiasari , S. Alamolhoda, Effects of iron oxide contents on photocatalytic performance of nanocomposites based on g‑C_3_N_4_, Scientific Reports, (2023) 13:6203.
